# A deep learning‐based 3D Prompt‐nnUnet model for automatic segmentation in brachytherapy of postoperative endometrial carcinoma

**DOI:** 10.1002/acm2.14371

**Published:** 2024-04-29

**Authors:** Xian Xue, Dazhu Liang, Kaiyue Wang, Jianwei Gao, Jingjing Ding, Fugen Zhou, Juan Xu, Hefeng Liu, Quanfu Sun, Ping Jiang, Laiyuan Tao, Wenzhao Shi, Jinsheng Cheng

**Affiliations:** ^1^ Secondary Standard Dosimetry Laboratory National Institute for Radiological Protection Chinese Center for Disease Control and Prevention (CDC) Beijing China; ^2^ Digital Health China Technologies Co., LTD Beijing China; ^3^ Department of Radiotherapy Peking University Third Hospital Beijing China; ^4^ Department of Radiotherapy Chinese People's Liberation Army (PLA) General Hospital Beijing China; ^5^ Department of Aero‐space Information Engineering Beihang University Beijing China

**Keywords:** autosegmentation of HR CTV or OAR, EC, HDR BT, Prompt‐nnUnet deep learning (DL) model

## Abstract

**Purpose:**

To create and evaluate a three‐dimensional (3D) Prompt‐nnUnet module that utilizes the prompts‐based model combined with 3D nnUnet for producing the rapid and consistent autosegmentation of high‐risk clinical target volume (HR CTV) and organ at risk (OAR) in high‐dose‐rate brachytherapy (HDR BT) for patients with postoperative endometrial carcinoma (EC).

**Methods and materials:**

On two experimental batches, a total of 321 computed tomography (CT) scans were obtained for HR CTV segmentation from 321 patients with EC, and 125 CT scans for OARs segmentation from 125 patients. The numbers of training/validation/test were 257/32/32 and 87/13/25 for HR CTV and OARs respectively. A novel comparison of the deep learning neural network 3D Prompt‐nnUnet and 3D nnUnet was applied for HR CTV and OARs segmentation. Three‐fold cross validation and several quantitative metrics were employed, including Dice similarity coefficient (DSC), Hausdorff distance (HD), 95th percentile of Hausdorff distance (HD95%), and intersection over union (IoU).

**Results:**

The Prompt‐nnUnet included two forms of parameters Predict‐Prompt (PP) and Label‐Prompt (LP), with the LP performing most similarly to the experienced radiation oncologist and outperforming the less experienced ones. During the testing phase, the mean DSC values for the LP were 0.96 ± 0.02, 0.91 ± 0.02, and 0.83 ± 0.07 for HR CTV, rectum and urethra, respectively. The mean HD values (mm) were 2.73 ± 0.95, 8.18 ± 4.84, and 2.11 ± 0.50, respectively. The mean HD95% values (mm) were 1.66 ± 1.11, 3.07 ± 0.94, and 1.35 ± 0.55, respectively. The mean IoUs were 0.92 ± 0.04, 0.84 ± 0.03, and 0.71 ± 0.09, respectively. A delineation time < 2.35 s per structure in the new model was observed, which was available to save clinician time.

**Conclusion:**

The Prompt‐nnUnet architecture, particularly the LP, was highly consistent with ground truth (GT) in HR CTV or OAR autosegmentation, reducing interobserver variability and shortening treatment time.

## 1 INTRODUCTION

Endometrial cancers (ECs), which primarily affect the epithelial lining, constitute the majority of uterine malignancies and rank as the sixth most common cancer among women.^1^, ^2^ Vaginal brachytherapy (VBT) compared to external beam radiation therapy (EBRT) has become an optimal postoperative radiotherapy for ECs, preventing local recurrence with lower toxicity and less impact on quality of life.^3^ A single channel high‐dose‐rate brachytherapy treatment plan combined with one vaginal cylinder was normally recommended for patients in the Postoperative Radiation Therapy in Endometrial Carcinoma (PORTEC) 4a trials.^4^ Contemporary BT employs adaptive treatment planning through 3D imaging, presenting benefits over traditional two‐dimensional (2D) imaging strategies.^5^ The accurate delineation of target volume using CT‐ or magnetic resonance (MR) images with a vaginal applicator in situ is so important that it can provide preferred data on dose distribution for treatments and minor acute radiation reactions for patients.^4^, ^6^ However, it is a generally challenging problem for clinicians to understand the guidelines and experience that achieve inter‐ and intra‐operator variability. Additionally, manual target contouring is a tedious and laborious work, typically taking more than 20 min,^7^, ^8^ which increases the heavy workload on limited time treatment planning. Therefore, computerized autosegmentation has been suggested as an alternative tool to promote the efficiency and repeatability of BT treatment.^9^, ^10^

Recently, Atlas‐based segmentation and Model‐based segmentation methods are two available modalities for auto‐delineating algorithms.^11^, ^12^ But the most significant shortcomings of them are limited to the specific anatomy and image contrast present in the atlas or training data, and require a large amount of representative atlas or annotated training data to achieve high accuracy.^13^, ^14^ One of alternative strategies to settle these undesired characteristics is a neural network technique, for example, convolutional neural networks (CNNs), with a particular emphasis on medical imaging applications.^15^ Although there are studies on malignancies of the female reproductive system, most of them have focused on EBRT, which limits their application to BT for gynecologic tumors. There are only 10 studies in this specific area that we could find, see Table 1. The DSC for HR CTV and rectum ranged between 0.65–0.97 and 0.74–0.97, respectively, with no segmentation of the urethra performed. Regarding adjuvant brachytherapy following radical hysterectomy, only two studies have been identified. With respect to postoperative target volume delineation, the shape of the target volume is regular for vaginal stump irradiation after radical hysterectomy, exhibits minimal applicator artifact, and presents lower model training complexity compared to non‐surgical patients.^16^, ^17^ Although these networks exhibit commendable performance, their utility for specific image segmentation tasks is frequently constrained. The necessity for task‐specific design and configuration demands meticulous fine‐tuning, as minor adjustments in hyperparameters can result in substantial performance disparities. A model optimized for a particular task is likely to underperform in different application scenarios.^18^, ^19^ The nnUnet framework, a specific variant of CNN, streamlines the process and automated setup by removing the manual tasks of preprocessing, network architecture design, training, and post processing, providing a pathway to wider implementation for automated image segmentation.^20^ Therefore, this paper proposed nnUnet as a standard baseline for HDR BT in EC patients.

**TABLE 1:** Summary of DL‐based autosegmentation results for targets and OARs in gynecological brachytherapy.

Studies	Objectives	DL models	Images	Structures	DSC	HD95% (mm)
Rodríguez Outeiral et al. (2023)^21^	CC	3D nnU‐Net	MRI	GTV	0.73	6.8
Yoganathan et al. (2022)^22^	CC	2.5DResNet, 2.5D InRN	MRI	GTV	0.60, 0.62	7.1, 6.8
				HR CTV	0.85, 0.85	5.0, 4.9
				IR CTV	0.75. 0.75	8.3, 7.96
				Rectum	0.74, 0.76	9.6, 8.2
				Sigmoid	0.64, 0.65	22, 20.4
				Small intestine	0.53, 0.54	26.7, 22.3
				Bladder	0.90, 0.90	6.5, 6.3
Zabihollahy et al. (2021)^23^	CC	3D Dense U‐Net (coarse‐to‐fine)	MRI	Rectum	0.88	2.24
				Sigmoid	0.80	3.28
				Bladder	0.94	2.89
Cao et al. (2021)^24^	CC	Dual‐path CNN	Preimplant MRI and postimplant CT	HR CTV	0.65 (small)^a^	7.34 (small)^a^
					0.79(medium)^b^	5.48(medium)^b^
					0.75 (large)^c^	6.21(large)^c^
Li et al. (2022)^18^	CC	nnUnet (2D U‐Net, 3D U‐Net and 3D‐Cascade U‐Net)	CT	HR CTV	0.84	7.42
				Rectum	0.83	7.58
				Bladder	0.94	3.50
Yan et al. (2023)^16^	Postoperative CC, EC	DANNs	CT	HR CTV	0.97	3.68
Wang et al. (2023)^17^	Postoperative CC	Modified CNN	CT	HR CTV	0.87	1.45^d^
				Rectum	0.86	2.52^d^
				Sigmoid	0.79	10.92^d^
				Small intestine	0.92	8.83^d^
				Bladder	0.94	4.52^d^
Mohammadi et al. (2021)^25^	CC	2D ResU‐Net	CT	Rectum	0.97	1.42
				Sigmoid	0.93	2.10
				Bladder	0.96	2.30
Jiang et al. (2021)^26^	CC	RefineNet	CT	HR CTV	0.86	6.01^d^
				Rectum	0.86	12.27^d^
				Sigmoid	0.66	98.41^d^
				Small intestine	0.56	68.12^d^
				Bladder	0.86	19.98^d^
Zhang et al. (2020)^27^	CC	3D DSD‐UNET	CT	HR CTV	0.83	8.10^d^
				Rectum	0.82	9.20^d^
				Sigmoid	0.65	19.6^d^
				Small intestine	0.80	27.8^d^
				Bladder	0.87	12.10^d^

Abbreviations: CC, cervical cancer; DANNs, domain‐adversarial neural networks; DSC, dice similarity coefficient; EC, endometrial cancer; GTV, gross tumor volume; HD 95%, 95th percentile of Hausdorff distance; HR CTV, high‐risk clinical target volume; InRN, InceptionResNetv; IR CTV, intermediate‐risk clinical target volume; OAR, organs‐at‐risk.

^a^ Small (< 20 cc).

^b^ Medium (20−40 cc).

^c^ Large tumor volume group (> 40 cc).

^d^ Indicates HD (mm).

Unlike conventional methods that necessitate training separate segmentation models for individual organs, the Segment Anything Model (SAM) empowers accurate segmentation of diverse organs, enabling a more versatile and generalized approach.^28^, ^29^ This pertains to prompt‐based segmentation, effectively partitioning the target using user‐provided cues, such as points, bounding boxes, or semantic representations and directives.^30^ Owing to its exceptional achievement across various visual recognition metrics, SAM has attracted significant attention as a valuable tool for tackling medical image contouring tasks. Zhang et al.^31^ carried out experiments with SAM for majority OARs achieving DSC > 0.7, the experimental findings demonstrated SAM's enhanced robustness and sustained precision in automated segmentation for radiotherapy. However, it demonstrates mediocre capability in certain medical delineation tasks. He et al.^32^ investigated the precision of SAM across 12 publicly available medical segmentation image samples encompassing diverse organs (brain, breast, chest, etc.), imaging techniques (radiography, pathology, endoscopy, and MRI and CT), and health statuses (normal, disease). The SAM, when used without specific medical image training, exhibited inferior achievements compared to U‐Net or other deep learning models. Chen et al.^33^ suggested that SAM executed strong overall performance with US images featuring well‐defined tissue structures, but it exhibited constrained efficacy when dealing with shadow noises and indistinct boundaries.

Segmentation precision is crucial in medical performance, serving as a foundation for many clinical applications. Prompt guided contouring has been proven to be more profitable than the limitations presented by SAM. This inspires us to develop an innovative architecture for prompt format segmentation. Our study objectives were to create a well‐established 3D nnUnet model with a prompt‐based component, termed 3D Prompt‐nnUnet, combining the advantages of nnUnet and the prompt capability for computer‐aided segmentation of HR CTV, and OAR. In addition, it was compared to manual contouring as the reference standard in postoperative EC brachytherapy. The new model has not only the basic segmentation capabilities, but also the ability to modify and correct the segmentation contour based on prompt information. It allowed faster and more accurate correction of delineation contour by the physician.

## 2 MATERIALS AND METHODS

**FIGURE 1:**
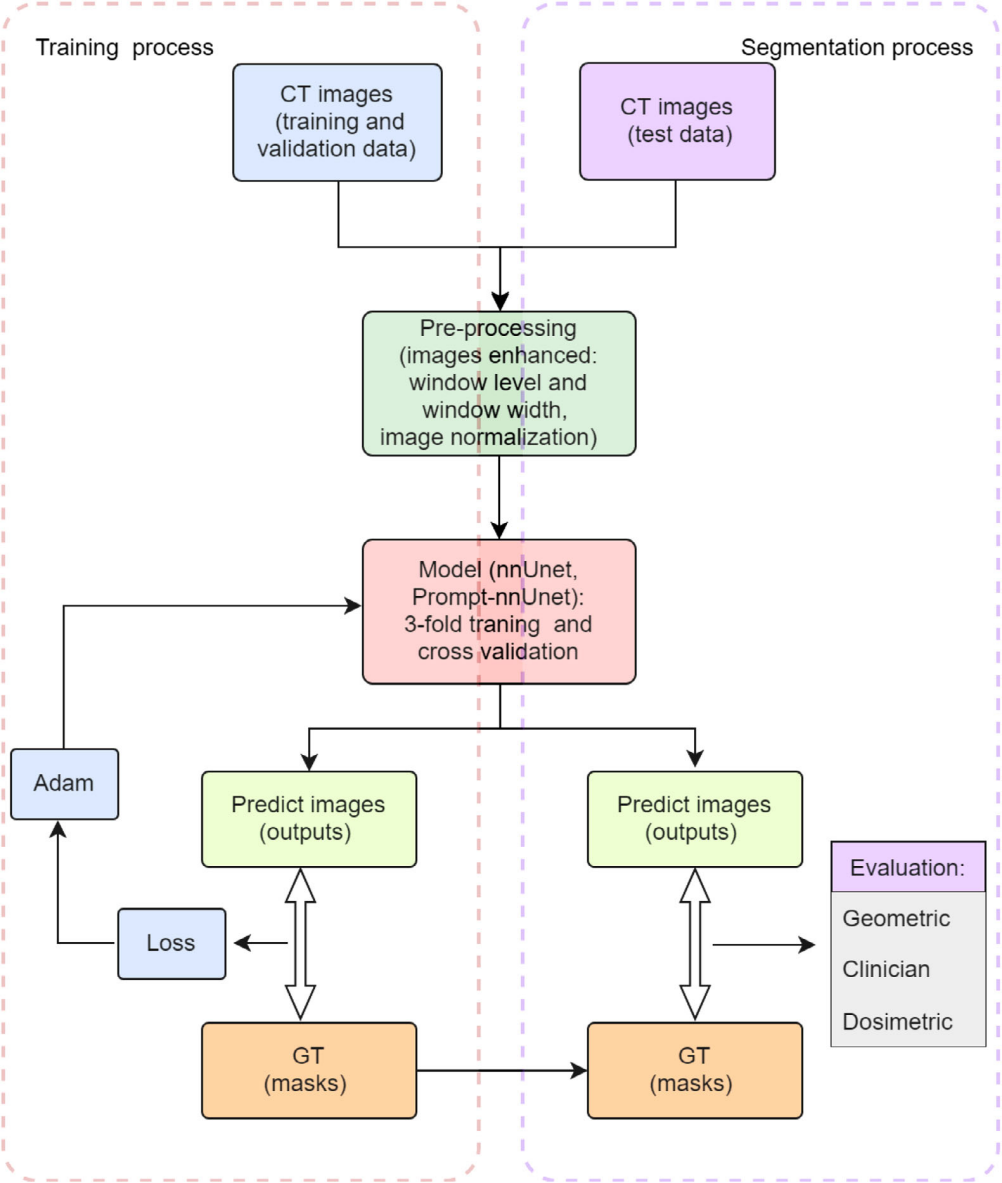
The workflow of training and test dataset for nnUnet and Prompt‐nnUnet.

The evaluation dataset comprised optimized images that excluded any personal information or private messages. Figure 1 was a flow diagram of our study.

### 2.1 Clinical data selection

The Research Review Board approved this retrospective study, conducted from November 2021 to October 2022. It included two batches of patients, with 321 in one batch and 125 in the other. The study excluded patients with recurrent vaginal stump and those with positive postoperative pathological stump findings. The inclusion criteria included EC patients who had undergone radical hysterectomy with high‐precision CT‐guided single‐channel VBT using an intravaginal applicator. Each patient contained 3–6 fractions (5.0 Gy/fraction), and each fraction required a rescanning CT. A total of 321 cases of 321 patients were considered for HR CTV, 257 were utilized in training, 32 in validation and 32 in testing. A total of 125 cases of 125 patients were for OAR involving the rectum and urethra with irregular or narrow shape, and 87, 13, and 25 cases were applied in training, validation and testing, respectively. A case here denotes a single fraction in the treatment.

In the CT scanning room, the bladder was infused with 80 mL of normal saline solution. All patients underwent scanning using a volumetric Brilliance Big Bore (Philips Healthcare, Best, the Netherlands) with a protocol of 140 kV and 300 mAs. The acquired images were subsequently transferred to a workstation and reconstructed using a matrix size of 512 × 512 and a slice thickness of 3 mm.

The contouring of HR CTV and OAR was performed by three senior radiation oncologists on Eclipse 15.5 (Varian Medical Systems, Inc. USA). The outline criteria for CTV were based on American Brachytherapy Society (ABS) recommendations^34^: the length was 3–5 cm from the tip of the applicator down (1/3 to 1/2 length of the vagina if the vaginal stump was short after surgery), and the thickness was 5 mm outside the applicator surface. The cylindrical‐like volumes of HR CTV in testing data points ranged from 10.14 to 55.94 cc with a diameter of 25−40 mm.

The outline criteria for OAR including the rectum and urethra were based on the Radiation Therapy Oncology Group (RTOG) outlining guideline consensus and the ICRU 89 report.^35^ The rectal contouring range was delineated from the anal sphincter to the rectum‐sigmoid colon junction of the outer wall. The urethral outline was identified with the aid of a Foley catheter from the internal urethral opening to the plane of the posterior inferior border of the pubic symphysis.

Each contour used as GT was adjusted and edited by a senior radiation oncologist, and finally reviewed and confirmed by another senior radiation oncologist.

### 2.2 Data preprocessing

Two batches of data from CT for CTV and OAR were selected to be outlined and labeled separately. The organs of interest were labeled 1 for CTV, and 1 or 2 for OARs, the other part as background.

We standardized the Hounsfield unit values of both types of CT images and limited the CT image intensity values of all scans to the range of [−166, 289] Hounsfield units for CTV and [−953,77] Hounsfield units for OARs to eliminate spurious details. The example of Original CT scans and their GT masks were in Figure 1 of the supplementary material.

### 2.3 Network architectures

The nnUnet adapted various image modalities, geometries, and dataset sizes without human intervention.^36^ Based on the U‐Net backbone, it offers three architectures: a 3D U‐Net cascade network (3D‐Cascade), a 2D U‐Net, and a 3D U‐Net training all pictures at full image resolution (3D‐Fullres). In our study, we directly choose 3D‐Fullres as nnUnet model for 3D image segmentation. To make nnUnet even more powerful, we have developed a 3D Prompt‐nnUnet framework that combines the above advantages of nnUnet and the prompt idea derived from SAM. The details were as follows.

**FIGURE 2:**
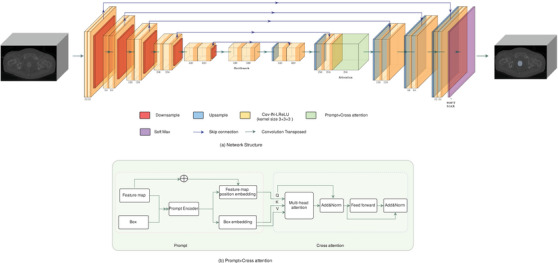
The framework of Prompt‐nnUnet (a) and process flow diagram for prompt part and cross attention component (b).

See Figure 2, one prompt‐based section was integrated into the 256‐channel nnUnet decoding path, which included two parts named prompt and cross attention parts.^37^ In the prompt part, the task of the prompt encoder (PE) is to provide a 3D coordinate (x, y, z) associated with the corresponding (256 in our model) dimensions. Consequently, the feature map was transmitted to PE and formed the position encoding of each point, which was combined with the feature map information to create queries (Q). For example, the feature map size was (10, 28, 28), the matrix of its three‐dimensional coordinates was denoted as (3, 10, 28, 28), the 256‐dimensional vector was generated by PE for each point, finally, the position encoding of each point and the feature map of the corresponding point were obtained as Q (256, 10, 28, 28). Bounding box, mainly using two coordinate end points of the body diagonal from the smallest outer cuboid of the organ of interest, was shown as “box" in the figure for prompt information including two types. If two points of vector information were predicted from the smallest outer cuboid of CTV or OAR structures from nnUnet, this was called a Predict‐ Prompt (PP). The prompt information was automatically imported from nnUnet. If two points information were from a manual mask, this was called a Label‐Prompt (LP). In our study, the labels have been delineated as GT, so we just used the label contouring to find the two points of the smallest cuboid automatically. However, physician merely marked the target's frame on the top, bottom, and largest layers of the 2D CT images to obtain the smallest cuboid of structures in practical scenarios. One of these two types of data in the box was also supplied to PE to create the spatial encoding for each position, where the position information of the points was Keys (K) and Values (V) formed (n, 256), where ‘n’ was the number of the smallest outer cuboid points. For example, ‘n’ was 2 for HR CTV, since the segmentation target was only one given 2 points for a box boundary, consequently ‘n’ was 4 for two OARs. Then Q, K, and V were transmitted to the cross‐attention part for the attention values (Avs),^37^ here there were eight multi‐heads in the cross‐attention part. The formula (Equation 1) is:

(1) $$\mathrm{Avs}\;=\;\mathrm{Softmax}\left(\frac{Q\times K^{T}}{\sqrt{256}}\right)\times V$$

Finally, the new Avs incorporating feature map and box position information were proceeded to nnUnet for next calculation. The Prompt made cross attention after the second up‐sampling of the original nnUnet decoder, maintaining the same size of the original feature map, and then operated the same as the nnUnet decoder.

### 2.4 Training and test processing and details

We trained two models nnUnet and Prompt‐nnUnet using HR CTV and OAR datasets respectively, HR CTV included one target, OAR two organs, rectum and urethra. A patch of size (40, 224, 224) was randomly cropped in the training phase. The input data were 3D images, and 3D convolution was used during training time. In training phase, three‐fold training was used for each architecture. In the prediction stage, sliding window with 0.5 overlap was used to maintain the same size as the original data.

In our study, these two of models were used for segmentation and comparing with each other, nnUnet was to segment the original data directly, Prompt‐nnUnet was to segment the target region after inputting the box prompt. There were two input prompt methods PP and LP mentioned above.

The PyTorch 1.12.0 and Python 3.9.7 framework were utilized to implement the neural network, which was performed on a workstation platform with an NVIDIA‐SMI 517.66 RTX A5000 16384MiB GPU. Due to memory constraints, a batch size of 2 was employed. The Adam optimizer with an initial learning rate of 0.1 was applied across the entire network. The loss function (Equation 2) was employed for effective 3D segmentation of volumetric data. The maximum number of epochs was determined to be 500 for HR CTV and 200 for OARs.

(2) $$\begin{array}{ccc} \mathrm{Loss}\; & = & W_{ce}\;\times \left(-y_{true}\log\left(y_{pred}\right)\right.\\ & & \left.-\;\left(1-y_{true}\right)\log\left(1-y_{pred}\right)\right)-W_{dice}\\ & & \times \;DSC\;\;DSC\;=\frac{2\times TP}{FN+TP+TP+FP}\; \end{array}$$

The loss function is the weighted sum of cross entropy loss and Dice loss, where $W_{ce}$ and $W_{dice}$ represent cross entropy loss and Dice loss weights, respectively, with a weighting ratio of 1:1. $y_{true}$ refers to the actual category, for example, 0 is the background of images, 1 is the foreground for HR CTV or OARs of EC in our model. $y_{pred}\;$ refers to the probability of the prediction being the category foreground. TP is the correct number of prediction pixels (positive sample, target, no background), FP is the number of pixels that are actually negative samples but predicted to be foreground, FN is the number of pixels that are actually positive samples but predicted to be background.

### 2.5 Quantitative assessment metrics

To obtain an overall measure of model performance, the mean and standard deviation (SD) values of assessment metrics as follows were calculated by comparing GT values. We used the DSC, HD, HD95%,^25^ and IoU^38^ to evaluate model performances, details in supplementary material.

### 2.6 Oncologist evaluation

The additional radiation oncologists (ROs) committee consisting of six oncologists classified as junior oncologist (JO) (two persons less than 5 years’ experience), intermediate oncologist (IO) (two persons more than 5 years’ and less than 10 years’ experience) and senior oncologist (SO) (two persons more than 10 years’ experience) was invited to implement the clinical subject evaluation.

There were three assessment methods for the evaluation. Each evaluation was performed by randomly selecting five cases from each batch of CTV and OAR cases. First, they were instructed to assess automatic contouring results based on a 4‐point scale criteria (details in the supplemental material): (1) Acceptable performance, (2) Slight modifications, (3) Significant modification, (4) Complete refusal. Second, three ROs independently contoured the HR CTV and OARs. The contours from GT, and ROs were then utilized to compute the DSC for assessing interoperator variability, and compare with DL‐models results. Third, the time taken for DL‐based automatic contouring was compared to the time spent on manual contouring by oncologists.

### 2.7 Dosimetric evaluation

To evaluate the impact of anticipated segmentation differences on dose‐volume parameters (DVP), the contours generated by PP, LP, nnUnet, and GT were incorporated into the treatment planning system. Treatment plans were developed using GT delineation (Oncentra Brachy 4.6.0. TPS. Elekta, Stockholm, Sweden). The external beam and the 2 Gy fraction equivalent dose (EQD2) in BT were taken into consideration during the plan. The absolute values were received from various parameters of DL models compared with the corresponding values obtained from GT to assess the discrepancies. The dose constraints for OARs and the prescribed dose adhered to the guidelines of the ABS for HDR‐BT. The HR CTV was designated a prescription dose of 5 Gy/fraction. An analysis was conducted on D90% (the minimum dose delivered to 90% of target volume), as well as volume at 100%, 150%, and 200% dose levels (V100%, V150%, V200%) on four types of CTV delineation. In the case of OARs, the assessment included the minimum doses received by 2, 1, and 0.1 cm^3^ volumes (D_2cc_, D_1cc_, D_0.1cc_), as well as the maximum dose (D_max_).^18^

### 2.8 Statistical evaluation

The statistical analysis was executed with IBM SPSS Statistics 21 software. The Mann–Whitney U test was used, with a *p*‐value < 0.05 indicating statistical significance.

## 3 RESULTS

### 3.1 Training processing evaluation

**FIGURE 3:**
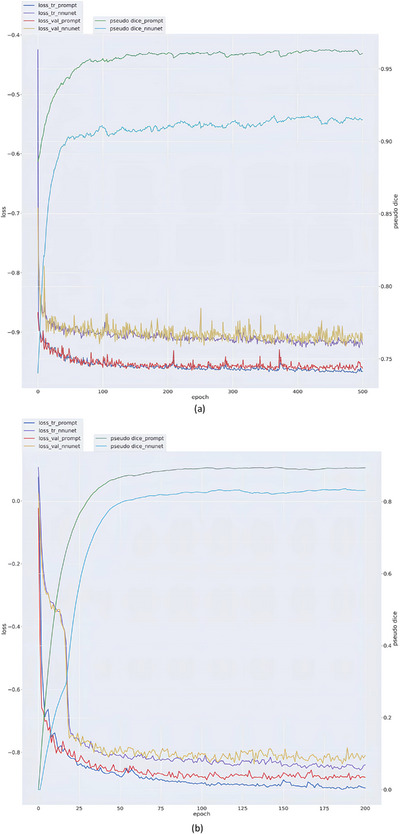
The average dice loss and mean DSC for validation dataset varied across different epochs for HR CTV (a) and OARs (b) in the3D LP nnUnet and nnUnet.

The Prompt‐nnUnet (including PP and LP) and nnUnet models were trained at the same time, approximately 46 h for HR CTV and 18 h for the OAR model (330 s for one epoch). The Prompt‐nnUnet created HR CTV or OARs within 2.35 s, however, the nnUnet created them within 33.64 s. The average dice loss and mean DSC for dataset depending on various epochs for HR CTV or OARs in the LP and nnUnet are given in Figure 3. The best mean DSC was approximately 0.97 at epoch 500 for HR CTV in LP, which was better than that of approximately 0.93 in nnUnet. Meanwhile, for OARs, there was 0.90 DSC achieved by the LP model compared to 0.84 in nnUnet.

### 3.2 Quantitative assessment

**FIGURE 4:**
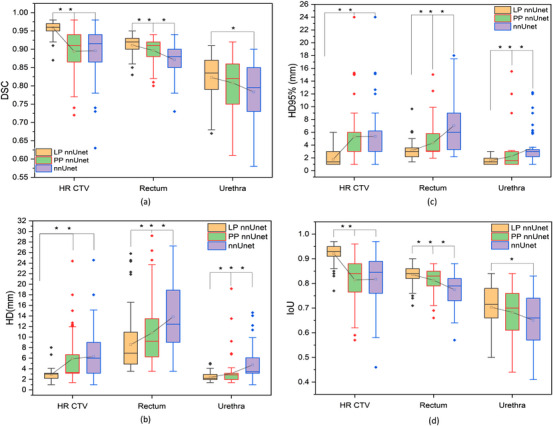
The auto‐segmentation performance of two network architectures was compared using evaluation metrics including DSC, HD, HD95%, and IoU. Lines, blocks, and diamonds indicated median values, mean values, and outliers, respectively. Significant differences between LP, PP, and nnUnet were denoted by an asterisk (*p < 0.05) in the results.

**FIGURE 5:**
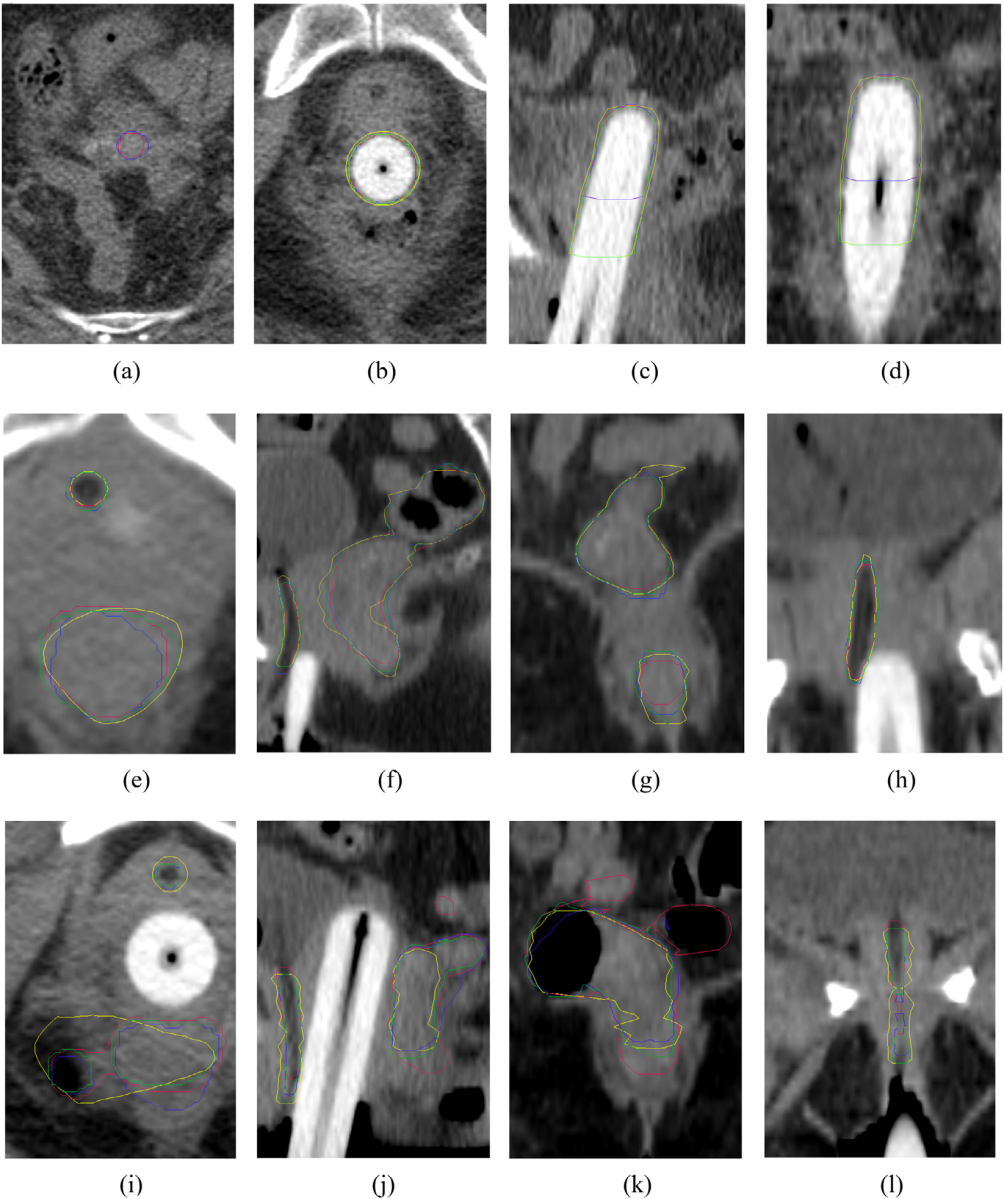
The comparison between the segmentation outcomes produced by the models (LP nnUnet: green, PP nnUnet: blue, nnUnet: red) and the manual delineation/GT (yellow) for HR CTV (a–d), rectum and urethra (e‐l). (a) showed the superior slice for HR CTV, indicating an extra slice segmentation of HR CTV for PP and nnUnet models; (b–d) indicated a small deviation between LP‐model segmentation from GT and a large deviation between PP and nnUnet from GT on inferior slices. (e–h) represented the better prediction example for the rectum and urethra of these models. (i–l) represented the erroneously predicted examples for the rectum and urethra.

In the testing program, Table 2 present the mean ± SD of DSC, HD, HD 95%, and IoU for HR CTV or OARs in DL models, while Figure 4 shows the boxplots of these values. The outliers number observed grounded on these subjects are in Table 1 of the supplemental materials. The comparison between the delineation results obtained from the models and clinician‐drawn contours was illustrated in Figure 5. All results showed that LP outperformed PP and nnUnet, and PP slightly outperformed nnUnet.

**TABLE 2:** Three‐fold cross validation outcomes of metric evaluations for autosegmentation of HR CTV and OAR in DL‐models.

			Geometric metrics			
Structure	Models	K‐fold	DSC	HD (mm)	HD95% (mm)	IoU
HR‐CTV	LP nnUnet	1‐fold	0.96 ± 0.02	2.73 ± 0.95	1.66 ± 1.11	0.92 ± 0.04
		2‐fold	0.96 ± 0.01	2.88 ± 1.01	1.85 ± 1.18	0.92 ± 0.03
		3‐fold	0.96 ± 0.02	3.07 ± 1.39	1.83 ± 0.97	0.92 ± 0.03
	PP nnUnet	1‐fold	0.91 ± 0.06	5.00 ± 4.18	4.57 ± 4.26	0.83 ± 0.09
		2‐fold	0.89 ± 0.05	6.01 ± 4.02	5.45 ± 3.68	0.81 ± 0.09
		3‐fold	0.88 ± 0.07	6.68 ± 4.44	5.92 ± 4.23	0.80 ± 0.10
	nnUnet	1‐fold	0.90 ± 0.07	5.52 ± 4.34	4.78 ± 4.27	0.83 ± 0.11
		2‐fold	0.90 ± 0.06	6.11 ± 3.83	5.28 ± 3.66	0.82 ± 0.09
		3‐fold	0.89 ± 0.07	7.24 ± 4.43	6.02 ± 4.12	0.80 ± 0.10
Rectum	LP nnUnet	1‐fold	0.91 ± 0.03	8.65 ± 5.24	3.17 ± 1.61	0.83 ± 0.05
		2‐fold	0.91 ± 0.02	8.18 ± 4.84	3.07 ± 0.94	0.84 ± 0.03
		3‐fold	0.91 ± 0.02	8.85 ± 5.87	3.21 ± 1.32	0.84 ± 0.04
	PP nnUnet	1‐fold	0.89 ± 0.04	10.48 ± 5.01	4.35 ± 2.50	0.81 ± 0.06
		2‐fold	0.89 ± 0.03	11.72 ± 6.94	4.96 ± 3.14	0.81 ± 0.05
		3‐fold	0.90 ± 0.02	10.16 ± 5.77	3.67 ± 1.44	0.82 ± 0.04
	nnUnet	1‐fold	0.87 ± 0.04	14.03 ± 6.12	7.06 ± 4.49	0.77 ± 0.07
		2‐fold	0.87 ± 0.05	14.99 ± 6.79	8.32 ± 4.89	0.77 ± 0.07
		3‐fold	0.88 ± 0.04	12.52 ± 5.44	5.75 ± 3.54	0.78 ± 0.06
Urethra	LP nnUnet	1‐fold	0.83 ± 0.07	2.11 ± 0.50	1.35 ± 0.55	0.71 ± 0.09
		2‐fold	0.82 ± 0.06	2.62 ± 0.95	1.75 ± 0.76	0.70 ± 0.08
		3‐fold	0.82 ± 0.07	2.38 ± 0.59	1.66 ± 0.62	0.70 ± 0.09
	PP nnUnet	1‐fold	0.81 ± 0.07	2.79 ± 2.28	2.01 ± 2.17	0.69 ± 0.09
		2‐fold	0.81 ± 0.06	3.25 ± 1.80	2.31 ± 1.62	0.68 ± 0.09
		3‐fold	0.80 ± 0.08	3.31 ± 3.30	2.46 ± 2.77	0.68 ± 0.10
	nnUnet	1‐fold	0.78 ± 0.09	4.43 ± 2.95	3.34 ± 2.28	0.65 ± 0.12
		2‐fold	0.78 ± 0.08	5.25 ± 3.32	4.03 ± 2.92	0.64 ± 0.10
		3‐fold	0.79 ± 0.09	4.51 ± 2.45	3.46 ± 2.02	0.66 ± 0.11

*Note*: All measurements were depicted as mean ± SD.

Abbreviations: DSC, Dice similarity coefficient; HD, Hausdorff distance; HD95%, Hausdorff distance 95th percentile; HR CTV, high‐risk clinical target volume; IoU, Intersection over Union; LP, Label‐Prompt; PP, Predict‐Prompt.

### 3.3 Oncologist evaluation

According to the 4‐point scale criteria for evaluating automatic contouring results described above, the proportions of DL‐based automatic delineation cases that received standards of 1, 2, and 3 in the three DL‐models were shown in Table 2 of the supplemental material. The LP nnUnet model showed improved efficacy in the DL‐based automatic segmentation investigation for HR CTV, with 53.3% of cases reaching acceptable performance levels. On the other hand, just 46.7% of cases required minor modifications, and none needed major revisions. Moderate results were shown by the PP nnUnet model, which performed satisfactory in 43.3% of cases, somewhat modified in 40%, and significantly modified in 16.7% of cases. In 36.7% of cases, the standard nnUnet model performed satisfactorily; in 43.3% of cases, it had minor alterations; and in 20% of cases, it underwent major modifications. The LP nnUnet model continued to be the most successful for the rectum, despite a 40% drop in acceptable performance rate and a noteworthy 10% of cases requiring major adjustment. All models showed decreased efficacy in the segmentation of the urethra; the basic nnUnet model had the lowest acceptable performance rate (20%) and the largest percentage of cases (33.3%) requiring substantial adjustments. In general, the LP nnUnet model fared better than the other models in terms of segmentations that were clinically acceptable without requiring a lot of changes, indicating its possible use in clinical settings.

To evaluate the potential clinical applicability of DL‐based autosegmentation, we conducted a comparative interoperator analysis in five cases of each HR CTV and OAR test batches. The results of this analysis were presented in Table 3.

**TABLE 3:** Comparative analysis of interoperator variability among ROs, DL‐models and GT in oncological assessments.

		DSC					
Structures	Models (vs. GT)	Case1	Case2	Case3	Case4	Case5	Mean ± SD
HR‐CTV	LP nnUnet	0.95	0.95	0.96	0.98	0.97	0.96 ± 0.01
	PP nnUnet	0.95	0.94	0.92	0.87	0.97	0.93 ± 0.04
	nnUnet	0.94	0.95	0.96	0.89	0.92	0.93 ± 0.03
	JO	0.79	0.80	0.88	0.79	0.84	0.82 ± 0.04
	IO	0.86	0.89	0.91	0.84	0.85	0.87 ± 0.03
	SO	0.94	0.92	0.90	0.88	0.89	0.90 ± 0.02
Rectum	LP nnUnet	0.83	0.87	0.89	0.90	0.93	0.88 ± 0.04
	PP nnUnet	0.82	0.82	0.89	0.90	0.92	0.87 ± 0.05
	nnUnet	0.80	0.79	0.91	0.88	0.93	0.86 ± 0.06
	JO	0.81	0.83	0.85	0.84	0.86	0.84 ± 0.02
	IO	0.81	0.85	0.87	0.88	0.87	0.85 ± 0.03
	SO	0.82	0.86	0.88	0.88	0.89	0.86 ± 0.03
Urethra	LP nnUnet	0.91	0.88	0.90	0.88	0.88	0.89 ± 0.01
	PP nnUnet	0.92	0.87	0.89	0.88	0.88	0.88 ± 0.02
	nnUnet	0.88	0.88	0.90	0.89	0.84	0.88 ± 0.02
	JO	0.73	0.86	0.78	0.80	0.86	0.81 ± 0.06
	IO	0.78	0.88	0.89	0.89	0.86	0.86 ± 0.05
	SO	0.82	0.88	0.89	0.90	0.89	0.88 ± 0.03

Abbreviations: DSC, dice similarity coefficient; GT, ground truth; HR CTV, high‐risk clinical target volume; IO, intermediate oncologist; JO, junior oncologist; LP, Label‐Prompt; PP, Predict‐Prompt; SO, senior oncologist; Models (vs. GT), the corresponding DSC was obtained by comparing the delineation obtained from the DL models and ROs (JO, IO, and SO) with the GT contours.

Regarding the efficiency of contouring, the duration of manual contouring performed by oncologists with varying qualifications was recorded and compared with that of contouring aided by DL models in another five cases of each HR CTV and OAR test datasets. LP and PP were two prompt types of Prompt nnUnet, and segmentation time was the same with each other. The minimal time spent in Prompt nnUnet for HR CTV was 1.71 ± 0.35s, while the used time were 32.29 ± 0.78s, 22.8 ± 2.59 min, 17.60 ± 2.07 min, and 11.60 ± 1.83 min for nnUnet, JO, IO, and SO, respectively. The DL‐models segmentation time was the same for the rectum and urethra, and the minimal time for Prompt nnUnet was 1.51 ± 0.10s. The details were in Table 3 of the supplemental material.

### 3.4 Dosimetric evaluation

To assess the precision of dose calculations, the DVP acquired from predicted outlines were compared against manually outlines/GT (Table 4). The prescribed dose administered to the patients was 5 Gy (20 cases for HR CTV, nine cases for OAR).^39^, ^40^ The DVP discrepancies were smaller for HR CTV and OAR in Promp‐nnUnet than in nnUnet.

**TABLE 4:** Dosimetric metrics results for HR CTV and OARs.

		Dosimetric metrics			
Structures	Models (vs. GT)	△D90%(cGy)	△V100%(cc)	△V150%(cc)	△V200%(cc)
HR CTV	LP nnUnet	23.05 ± 23.93	0.92 ± 0.95	0.05 ± 0.10	0.02 ± 0.04
	PP nnUnet	43.28 ± 42.01	2.16 ± 3.33	0.78 ± 1.61	0.40 ± 1.04
	nnUnet	44.83 ± 44.21	2.47 ± 3.47	1.00 ± 1.77	0.77 ± 1.84
		△D_2cc_(cGy)	△D_1cc_(cGy)	△D_0.1cc_(cGy)	△V(cc)
Rectum	LP nnUnet	12.87 ± 11.08	14.40 ± 12.28	23.41 ± 20.44	3.42 ± 3.04
	PP nnUnet	16.28 ± 9.24	16.38 ± 10.63	24.30 ± 20.00	5.62 ± 4.69
	nnUnet	16.63 ± 9.83	17.07 ± 10.58	26.23 ± 22.72	9.80 ± 8.53
		△D_1cc_(cGy)	△D_0.1cc_(cGy)	△D_max_(cGy)	△V(cc)
Urethra	LP nnUnet	22.22 ± 12.03	8.92 ± 6.88	12.22 ± 13.94	0.48 ± 0.53
	PP nnUnet	26.39 ± 17.02	10.50 ± 5.65	13.22 ± 13.05	0.50 ± 0.52
	nnUnet	31.77 ± 23.51	10.68 ± 6.26	17.78 ± 17.87	0.52 ± 0.60

*Note*: All measurements were depicted as mean ± SD.

Abbreviations: HR CTV, high‐risk clinical target volume; LP, Label‐Prompt; PP, Predict‐Prompt; △D, absolute dose values of dosimetric differences between DL and GT; △V, absolute volume values of volume metric differences between DL and GT. Models (vs. GT): The dosimetric parameters of the DL model were compared with those of the GT profile.

## 4 DISCUSSION

Accurate delineation of CTV and OARs is of utmost importance in VBT. However, manual delineation is susceptible to observer variability, as it relies on different operators with varying levels of experience. Furthermore, this manual process is time‐consuming. The introduction of autocontouring for CTV or OARs in VBT is greatly desirable as it offers several advantages. It can help reduce the time gap between implantation and treatment, and minimize the variability associated with manual structure delineation. Accurate CTV and OARs delineation provides essential data on dose distribution for treatment and lessens the possibility of acute radiation toxicity in patients. The objective of this study was to create a new 3D Prompt‐nnUnet neural network architectures comparison with the conventional 3D nnUnet framework to perform autosegmentation of HR CTV and OARs in VBT. It was observed that the contours generated by Prompt‐nnUnet model, especially LP, exhibited a high degree of similarity/consistency with the manual contours created by experienced radiation oncologists, while also significantly reducing the time required for HR CTV or OARs delineation. Compared to the 3D nnUnet, this new model showed superior performance and a more streamlined workflow.

Our observations revealed that the results from three‐fold cross‐validation were comparable. LP predicted structures with the best DSCs of 0.96 ± 0.01 for HR CTV, 0.91 ± 0.02 for the rectum, and 0.83 ± 0.07 for the urethra. PP received the best DSCs of 0.91 ± 0.06, 0.90 ± 0.02, and 0.81 ± 0.06, while nnUnet achieved the best DSCs of 0.90 ± 0.06, 0.88 ± 0.04, and 0.79 ± 0.09, respectively. Meanwhile, the results were remarkably statically significant among all the metrics for LP compared to nnUnet. This result indicated that Prompt‐nnUnet particularly LP improved the nnUnet performance metrics, demonstrated the superiority of this model. We found that the box prompt information was so important that it directly affected the precision of automatic segmentation, so when it was from manual labeling location details such as LP data, the results showed better than others. Among all the structures, HR CTV exhibited the optimal DSC score and superior execution in other quantitative metrics. The primary reason for the inferior results observed in the rectum and urethra, as compared to the HR CTV, was primarily due to the geometrical variations in the OARs, particularly the urethra's narrow shape.^41^ There were significantly greater variations near tumors. Additionally, the information provided at the two points of the box prompt was insufficient for accurately addressing these complex organs. The dosimetric assessment demonstrated a consistent pattern with the segmentation results, showing that the Prompt‐nnUnet outperformed the 3D nnUnet model. This superiority resulted in minimized errors in the estimated absorbed dose within the structures.

See Table 1, there are only two papers studied in postoperative CC or EC. Yan et al.^16^ investigated HR CTV for postoperative VBT patients with the use of the DANN model. The median DSC and HD95% values were 0.97 and 3.68 mm in the DANNs with 90 patient CT images, compared to our LP model 0.96 and 1.38 mm. LP model demonstrated superior performance in terms of HD95% and 1% inferiority to DSC. It indicated that the LP model for boundary overlap of the segmented organs was better. This might be due to the accuracy of the coordinate information of HR CTV in the LP model and there could be variations in the training data causing the lower DSC values. Wang et al.^17^ introduced modified CNN for the automatic contouring of HR CTV and OARs in 60 CT scans of patients diagnosed with postoperative cervical cancer undergoing BT. The outcomes of our study demonstrated superior performance in DSC for HR‐CTV and rectum. It is difficult to define which model is superior without evaluating its performance on a specific task or dataset. However, the better results of the Prompt‐nnUnet model could be influenced by the particular demands of the task, the size and quality of the dataset, or other factors such as computational resources and training time.^42^ Mohammadi et al.^25^ used 2D ResU‐Net for OARs automatic delineation, involving 113 patients with locally‐advanced cervical cancer, 83 cases allocated for training and 30 for testing. The DSC and HD95% for the rectum were 0.97 and 1.42 mm, respectively, which appeared to be better results than ours (0.91 and 3.07 mm) for the 3D LP model with the numbers of 87 training, 13 validations, and 25 tests. These observed discrepancies in our study about postoperative EC might be attributed to two primary factors. First, we used a different dataset from Mohammadi et al.’s, the volume of interest was more complicated and variable. A radical hysterectomy may cause modifications to the rectum's morphology by altering the spatial relationship between it and adjacent tissues. These modifications not only deviate from the typical presentation seen in cervical cancer patients but also pose increased challenges in accurately defining the boundaries of the rectum.^43^, ^44^, ^45^ Second, although 3D models offer advantages in capturing volumetric spatial information compared to 2D counterparts, they have inherent limitations. The transition to higher dimensionality requires greater computational resources, leads to prolonged inference times, and distorts optimal performance. Additionally, the complexity associated with a larger parameter space increases the risk of overfitting, especially when dealing with limited datasets.^46^, ^47^ Thus we obtained a relatively lower DSC value for the rectum. In terms of other direct comparisons, the DSC value of Prompt‐nnUnet for automatic delineation in our study proved to be more advantageous against analogous findings from previous research.^48^, ^49^, ^50^, ^51^

In this study, the nnUnet model had fewer outliers in the automatic delineation contours than the Prompt‐nnUnet framework. Additionally, the majority of the mean/median values for the metric evaluation exhibited superiority over those of the nnUNet model. Unlike most studies that use one or few metrics for evaluation, we analyzed multiple metrics to assess the performance of each model. Our analysis showed that the LP model had approximately 20% or 30% more direct acceptance of use compared to PP or nnUnet. This could be attributed to the fact that the LP model produced more stable predicted contours that closely resembled the manual contours.

The research compared the accuracy of manual contouring by oncologists with varying levels of experience to DL‐based automatic segmentation for HR CTV or OARs. The results indicated that the automatic segmentation approach was comparable to the manual contours created by SO and outperformed those produced by JO and IO. JO and IO can utilize DL tools to improve their contouring consistency and minimize the modifying contours time. The study revealed that the DL‐based automatic segmentation method required less than 34 s, especially it took less than 1.8 s when based on the Prompt‐nnUnet model which required the least time in both models, while manual contouring took between 10 and 26 min in Table 3 of the supplementary material. The superiority of the automatic segmentation algorithm over manual contouring was apparent. However, the study had a limited data source and further research is necessary to determine the effectiveness of DL‐assisted contouring in larger populations and different diseases.

While our deep learning algorithm showed promising results in HR CTV, rectum and urethra contouring, it is important to acknowledge the inherent limitations of our study. These include challenges in conducting at a unique center, which may limit the general applicability of our outcomes to other clinical settings with varying patient populations, treatment volumes, and imaging protocols. Additionally, the use of different types of applicators and cancer types may introduce variability in results, making it difficult to directly compare our findings with those reported in other studies. Moreover, in our study, the information from the box frame encoder was insufficient for the delineation of complicated organs, rectum or urethra, leading to unsatisfactory geometric results. Finally, in recent study of cervical cancer segmentation using this model, we encountered a patient with a prosthesis. In the initial training phase, the model mistakenly classified prosthetic regions as tumorous tissue. This error was subsequently corrected to align with expected measurements. This instance highlights the necessity of refining our algorithm's differentiation capabilities to accurately discern between closely related target zones. Despite these limitations, our deep learning algorithm demonstrated superior performance when compared to other commonly used contouring approaches, such as atlas‐based^52^ and model‐based methods.^53^ Therefore, we believe that further investigation and refinement of our deep learning algorithm has the potential to enhance the precision and efficiency of HR CTV and OARs contouring in clinical settings.

## 5 CONCLUSION

In short, our research has confirmed that using DL technology for the autocontouring of HR CTV and OAR in VBT is both feasible and effective. The 3D Prompt‐nnUnet model outperformed the 3D nnUnet model in terms of quantitative assessment metrics for segmenting endometrial cancer. We have demonstrated that the DL tools can produce contouring results that are comparable in accuracy to those obtained by experienced senior oncologists, and superior to those provided by junior and intermediate oncologists. Furthermore, the use of LP can significantly enhance the contouring accuracy of junior and intermediate oncologists, while also reducing the contouring time required for all oncologists. Therefore, the DL tool holds great promise for improving the therapeutic outcomes of PORTEC.

## AUTHOR CONTRIBUTIONS

The study was conceptualized by Xian Xue, Fugen Zhou, and Quanfu Sun. Dazhu Liang, Jianwei Gao, Juan Xu, and Xian Xue performed data analysis. Kaiyue Wang, Jingjing Ding, and Ping Jiang provided interpretation of the clinical results. Xian Xue was responsible for writing the paper, while all authors participated in result discussions and manuscript revisions.

## CONFLICT OF INTEREST STATEMENT

The authors have no conflict of interest to disclose.

## Supporting information

Supporting Information

## Data Availability

Research data are not available at this time.
