# Supplementary material for: A deep learning‐based 3D Prompt‐nnUnet model for automatic segmentation in brachytherapy of postoperative endometrial carcinoma
Source: J Appl Clin Med Phys. 2024 Apr 29;25(7):e14371. doi: 10.1002/acm2.14371 (PMC11244685; doi:10.1002/acm2.14371)
Supplement: Supplementary file 1 — Supporting Information [file ACM2-25-e14371-s001.docx]

**Quantitative assessment metrics:**

To obtain an overall measure of model performance, the mean and standard deviation (SD) values of assessment metrics as follows were calculated by comparing ground truth values with deep learning results obtained from each 3D volume across testing patients.

1. The Dice similarity coefficient (DSC ) defined as:

$DSC=2\times\frac{A\cap B}{\left| A \right|+\left| B \right|}$ (1)

When referring to manual contouring and segmentation results as *A* and *B*, the DSC values provide a measure of overlap between the two, ranging from 0 to 1 where 1 indicates perfect agreement.

1. The Hausdorff distance (HD) is a measure of the similarity between two sets of points in a metric space. The Hausdorff distance 95th percentile (HD95%) is a statistical measure that represents the value below which 95% of the Hausdorff distances fall. It is commonly used to summarize the distribution of Hausdorff distances between two sets of points in a metric space. Both are measured in millimeters.

$\mathrm{HD}\left( A,B \right)=\max_{a\in A}\left\{ {min}_{b\in B}\left\{ d\left( a,b \right) \right\} \right\}$ (2)

Where a and b are points in the sets representing the ground truth and model prediction respectively. The Euclidean distance d is a defined as the maximum distance between any two points. A smaller Hausdorff distance value indicates that they are more similar or have more overlap.

1. Compute average Intersection over Union (IoU) measures the overlap between the predicted bounding box and the ground truth bounding box of an object in an image.

$\mathrm{IoU}\mathbf{=}\frac{A\cap B}{A\cup B}$ (3)

Where A and B denote the predicted bounding box and the ground truth bounding box respectively.

IoU is expressed as a value between 0 and 1, where a value of 0 means no overlap between the predicted and ground truth bounding boxes, and a value of 1 means perfect overlap between the two boxes. In general, a higher IoU indicates better object detection performance.

**4-point scale criteria:**

a 4-point scale criteria for estimating the therapeutic impact in consensus: 1. Acceptable performance- The segmentation results can be considered satisfactory and are deemed suitable for further treatment applications, 2. Slight modifications-delineation still required to address a few areas that need improvement, while these changes are not expected to have any significant clinical impact if left uncorrected, 3. Significant modification-automatic segmentation models can’t be used directly without significant contour corrections, 4. Complete refusal-the perceived errors exist in segmentation that need to be redrawn.

**The outliers on the DSC, HD, HD 95%, and IoU:**

**Table 1**. Outlier data points identified based on DCS, HD, HD95%, MSE and IoU

| Structure | Models | Number of patients with outlier values included | | | |
| --- | --- | --- | --- | --- | --- |
|  |  | DCS | HD | HD95% | IoU |
| HR CTV | LP nnUnet | 2 | 2 | 0 | 4 |
|  | PP nnUnet | 2 | 6 | 5 | 2 |
|  | nnUnet | 3 | 2 | 5 | 1 |
| Rectum | LP nnUnet | 2 | 5 | 3 | 3 |
|  | PP nnUnet | 2 | 3 | 2 | 2 |
|  | nnUnet | 1 | 0 | 1 | 1 |
| Urethra | LP nnUnet | 1 | 2 | 0 | 0 |
|  | PP nnUnet | 0 | 5 | 3 | 0 |
|  | nnUnet | 0 | 4 | 8 | 0 |

HR CTV: high-risk clinical target volume; LP: Label-Prompt; PP: Predict-Prompt; DSC: Dice similarity coefficient; HD: Hausdorff distance; HD95%; Hausdorff distance 95th percentile; IoU: Intersection over Union.

**Oncologist evaluation (**4-point scale criteria) **Results:**

**Table 2.** The proportion of DL-based automatic segmentation cases scored as 3 levels in DL-models

| Structures | Models | Acceptable performance (%) | Slight modifications (%) | Significant modification (%) |
| --- | --- | --- | --- | --- |
| HR CTV | LP nnUnet | 53.3 | 46.7 | 0 |
|  | PP nnUnet | 43.3 | 40.0 | 16.7 |
|  | nnUnet | 36.7 | 43.3 | 20.0 |
| Rectum | LP nnUnet | 40.0 | 50.0 | 10.0 |
|  | PP nnUnet | 36.7 | 40.0 | 23.3 |
|  | nnUnet | 30.0 | 43.3 | 26.7 |
| Urethra | LP nnUnet | 30.0 | 43.3 | 26.7 |
|  | PP nnUnet | 23.3 | 46.7 | 30.0 |
|  | nnUnet | 20.0 | 46.7 | 33.3 |

HR CTV: high-risk clinical target volume; LP: Label-Prompt; PP: Predict-Prompt.

**Time taken for DL-based automatic contouring and manual delineation:**

**Table 3.** Average time commitment for oncologist with different levels of expertise to perform manual contouring and DL-based auto-segmentation for segmentation purposes

| Models | Time(mean$\pm$SD) |  |  |
| --- | --- | --- | --- |
|  | HR CTV | Rectum | Urethra |
| Prompt nnUnet(including PP and LP) | 1.71$\pm0.35$s | 1.51$\pm0.10$s | 1.51$\pm0.10$s |
| nnUnet | 32.29$\pm0.78$s | 32.05$\pm0.02$s | 32.05$\pm0.02$s |
| JO | 22.8$0\pm2.59$min | 22.58$\pm$2.90min | 17.24$\pm$1.28min |
| IO | 17.6$0\pm2.07$min | 19.4$0\pm$3.05 min | 14.08$\pm$1.69min |
| SO | 11.6$0\pm$1.83min | 16.80$\pm2.59$min | 11.84$\pm$0.94min |

JO: junior oncologist; IO: intermediate oncologist; SO: senior oncologists. PP: Predict-Prompt; LP: Label-Prompt.

**Original CT scans and their ground truth (GT) masks:**

| 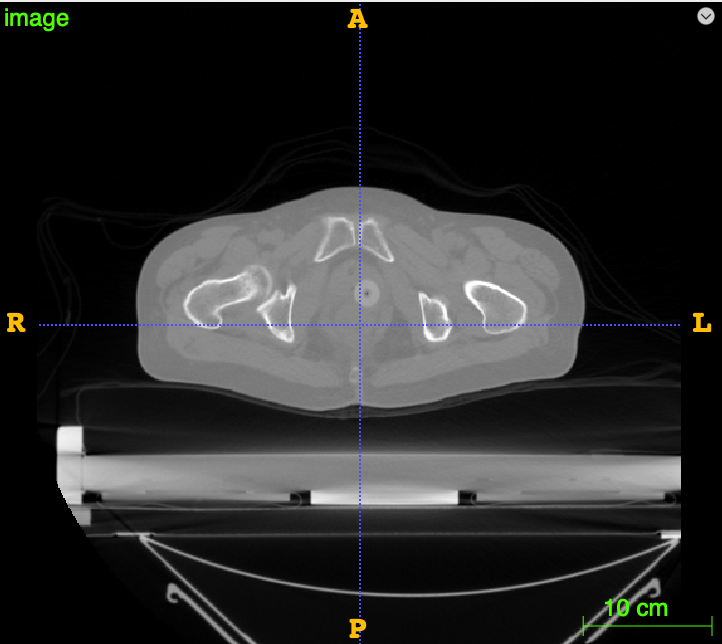  (a) | 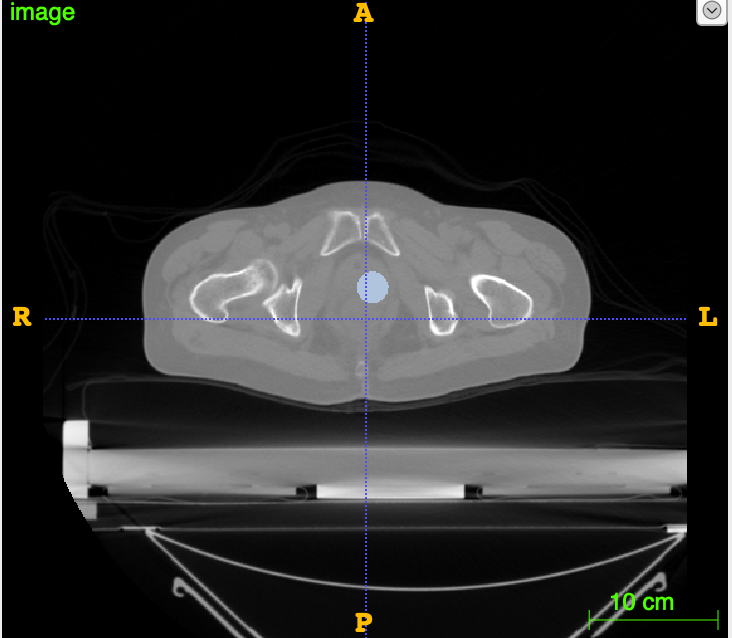  (b) |
| --- | --- |
| 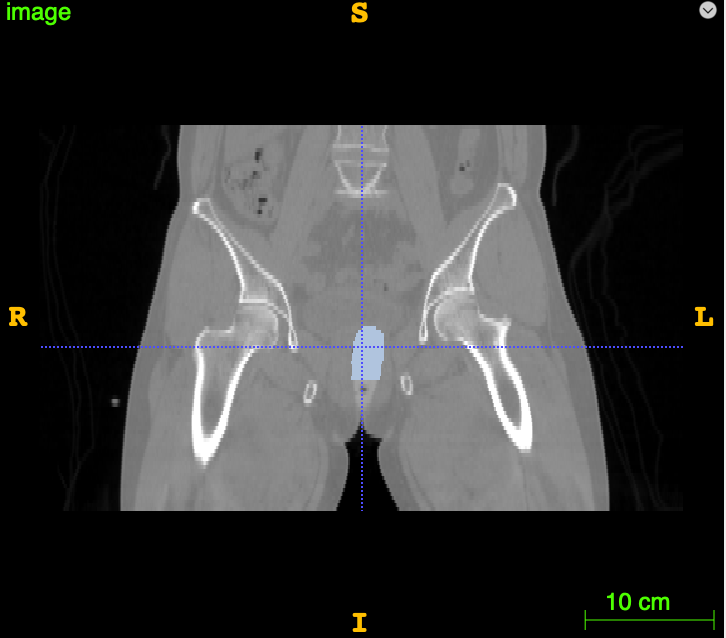(c) | 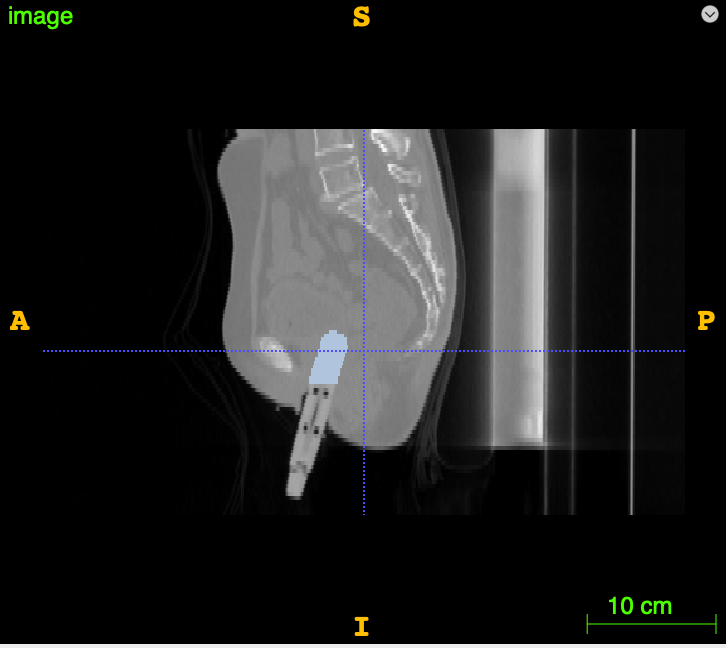(d) |
| 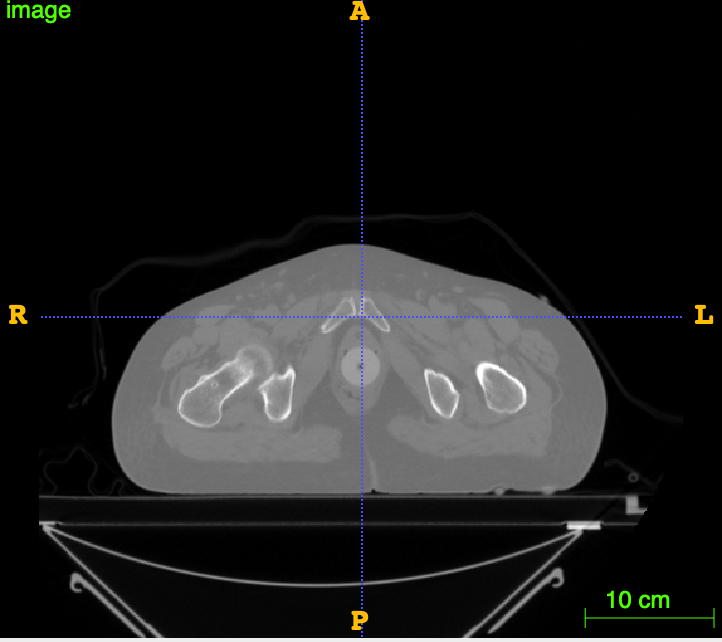(e) | 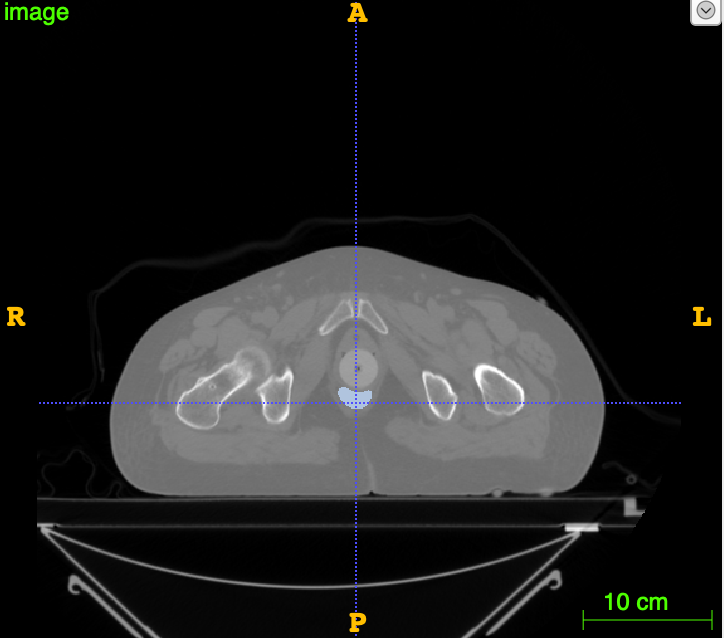(f) |
| 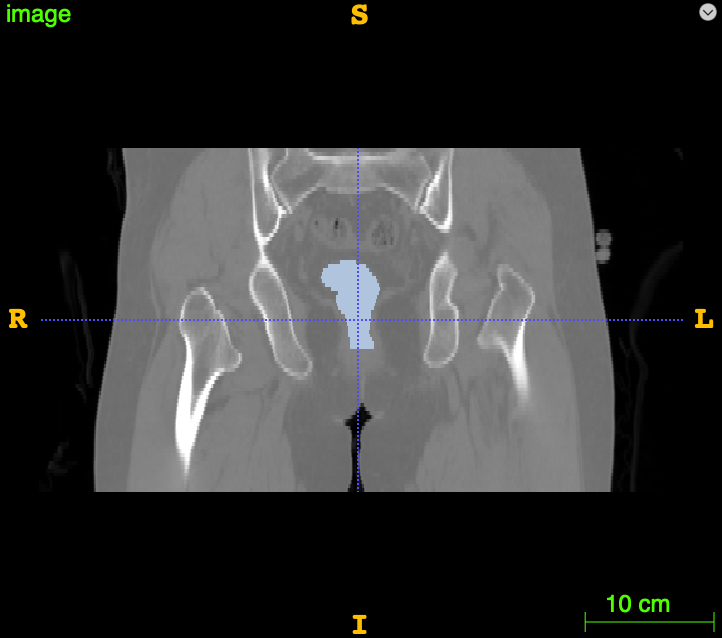(g) | 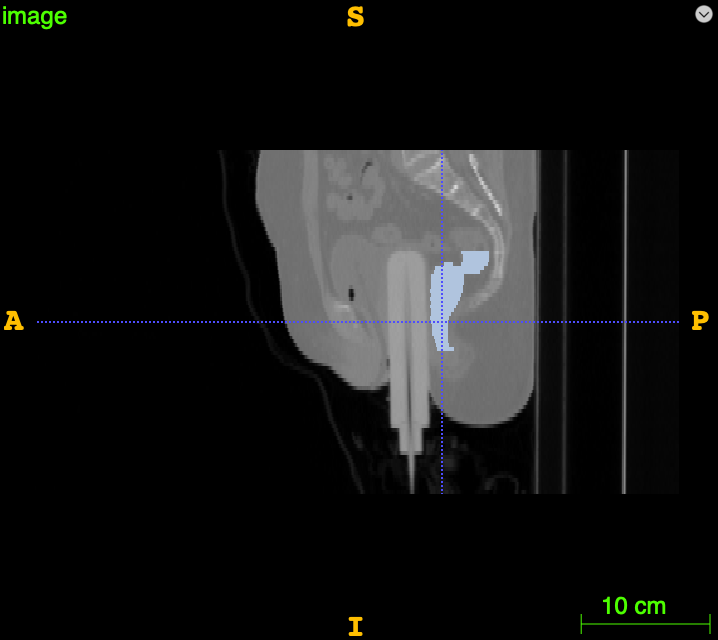(h) |
| 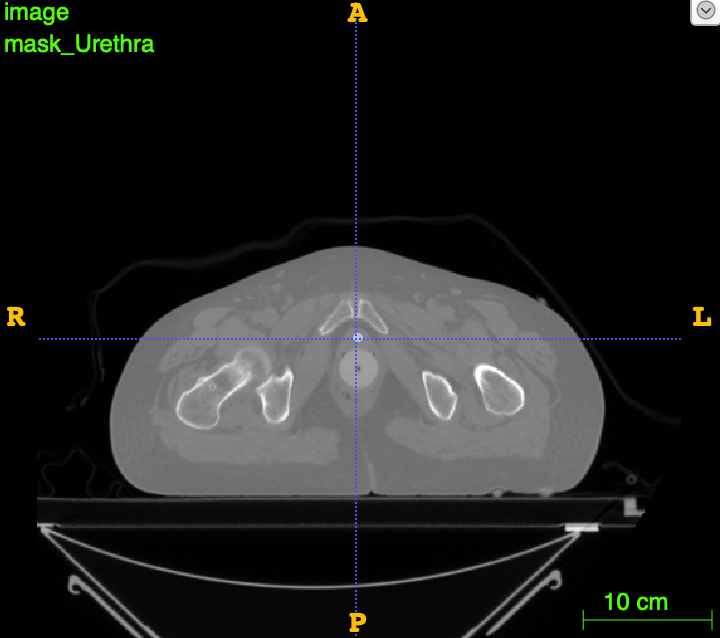(i) | 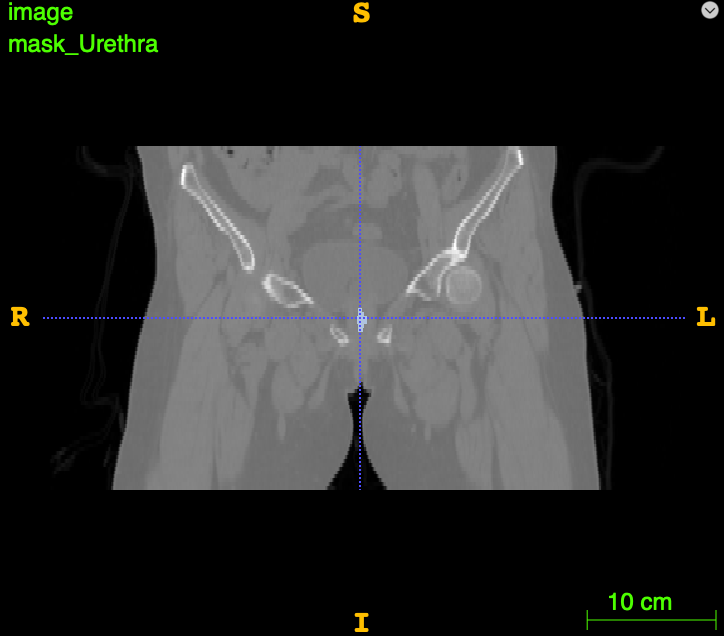(j) |
| 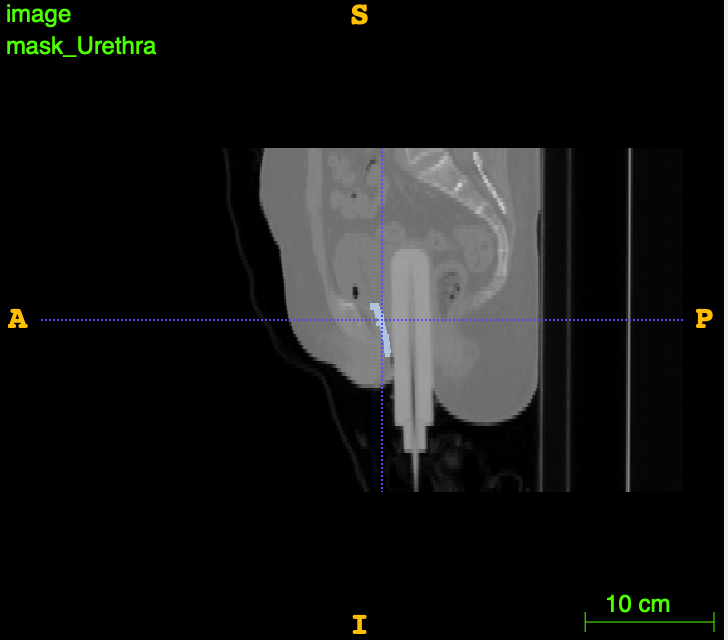(h) |  |

**Fig. 1** (a)-(d) were HR CTV original CT image and GT masks (blue), (e)-(h) were OARs (rectum and urethra) original CT image and GT masks (blue).
